# Efficacy of 5‐Hz Transcranial Magnetic Stimulation Over the Left Dorsolateral Prefrontal Cortex in Improving Cognitive Function During the Recovery Phase of Stroke

**DOI:** 10.1002/brb3.71402

**Published:** 2026-04-14

**Authors:** Hui‐xian Yu, Qian Diao, Yu‐juan Han, Bo Wei, Pei Dai

**Affiliations:** ^1^ Department of Rehabilitation Medicine, Beijing Tian Tan Hospital Capital Medical University Beijing China; ^2^ Tiantan Xiaotangshan Rehabilitation Center Beijing Xiaotangshan Hospital Beijing China

**Keywords:** cognitive impairment, dorsolateral prefrontal cortex, high‐frequency, stroke, transcranial magnetic stimulation

## Abstract

**Objective:**

Here, we aimed to explore the efficacy of 5‐Hz transcranial magnetic stimulation (TMS) over the left dorsolateral prefrontal cortex (DLPFC) in improving cognitive function during the recovery phase of stroke.

**Method:**

This was a retrospective study. A total of 33 patients were enrolled, of whom 13 received 5‐Hz TMS treatment and 20 did not. Cognitive functioning was assessed before and after treatment.

**Results:**

Montreal Cognitive Assessment (MoCA) scores increased significantly after treatment in the TMS group compared with the control group (*p* = 0.0359). MoCA scores in the TMS group also increased significantly after treatment compared with before (*p* < 0.0001). In the TMS group (including the left‐side lesions subgroup and the right‐side lesions subgroup), MoCA scores increased only in patients who had received left‐side lesions (*p* = 0.0156). However, we did not find any significant between‐group differences in MoCA scores after treatment.

**Conclusion:**

High‐frequency (5 Hz) TMS applied to the left DLPFC significantly enhances overall cognitive functioning in patients who are recovering from strokes. Interestingly, we found that when 5‐Hz TMS was applied to the left DLPFC, its effects were similar regardless of whether the lesion was ipsilateral to the stimulation site or not.

## Graphical abstract

A total of 51 patients were initially screened, of whom 33 were ultimately enrolled. These participants were divided into two groups based on whether they receivedTMS therapy: the TMS group (*n* = 13) and the control group (*n* = 20). Additionally, the TMS group was further stratified into subgroups with left‐sided lesions (*n* = 7) and right‐sided lesions (*n* = 6). MoCA was performed both before and after treatment in all cases.

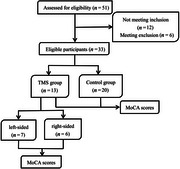

## 1 Introduction

The incidence of cognitive impairment has been progressively increasing and has been doing so in correlation with annual rises in stroke prevalence. Poststroke cognitive impairment (PSCI) refers to a series of syndromes that meet the diagnostic criteria for cognitive impairment within the first 6 months after a stroke (Rost et al. 2022). About 33% of stroke patients demonstrate significant cognitive impairment within 30–90 days of stroke onset (Gorelick and Nyenhuis 2015, Ayehu et al. 2023). Stroke‐associated cognitive impairment can affect many domains, including selective attention, executive functioning, and memory (Gallucci et al. 2024). Compared with conventional cognitive function training and pharmacologic interventions, noninvasive brain stimulation (NIBS) techniques have gained increasing attention due to their potential to facilitate cognitive recovery.

NIBS drives neural function recovery by modulating brain excitability (Kemps et al. 2022). Specifically, transcranial magnetic stimulation (TMS) applies pulsed magnetic fields to cortical and subcortical tissue, inducing brief but robust currents that depolarize local neurons. This process alters cortical activity and may lead to long‐lasting changes in neural plasticity. High‐frequency stimulation (≥5 Hz) appears to enhance the excitability of local neurons, while low‐frequency stimulation (≤1 Hz) has inhibitory effects (Klomjai et al. 2015). Recent animal studies have suggested that both low‐frequency (1 Hz) and high‐frequency (5 Hz) TMS can mitigate neuronal damage and preserve the excitation/inhibition balance. However, high‐frequency TMS is better than low‐frequency TMS at promoting functional hyperemia recovery and increasing neurotransmitter levels at postsynaptic sites (Liu et al. 2024). In a recent network meta‐analysis, high‐frequency TMS was recognized as the most effective way to enhance overall cognitive functioning (Yan et al. 2024). Additionally, most previous TMS studies have used the left dorsolateral prefrontal cortex (DLPFC) as their primary stimulation site.

Over the past several years, an increasing number of studies have addressed the selection of TMS stimulation sites in poststroke patients. Specifically, they have explored whether TMS should target the affected or healthy side of the brain, as well as the utility of low‐frequency versus high‐frequency treatments. High‐frequency TMS over the left DLPFC (Zheng et al. 2020), low‐frequency TMS over the healthy side of the brain (Li et al. 2021), high‐frequency TMS over the affected side of the brain (Cha et al. 2022), and bilateral high‐frequency TMS (Xu et al. 2022) all appear to enhance cognitive functioning. In most studies, however, high‐frequency TMS has been applied at a frequency of 10 Hz or higher. Here, we aimed to explore the efficacy of 5‐Hz TMS over the left DLPFC in improving cognitive functioning during subacute poststroke recovery. We then investigated whether the therapeutic outcomes differed when left‐DLPFC TMS was applied, based on whether the stimulation site was ipsilateral or contralateral to the stroke lesion.

## 2 Materials and Methods

### 2.1 Study Population

This was a retrospective study. A total of 33 patients with strokes who were hospitalized at the Beijing Xiaotangshan Hospital Tiantan Xiaotangshan Rehabilitation Center between June 2024 and December 2024 were enrolled.

Thirteen patients underwent rTMS in conjunction with routine rehabilitation, while 20 patients received only routine rehabilitation.

The inclusion criteria were as follows: (1) patients with strokes conclusively diagnosed on either CT or MRI (including cerebral hemorrhages and cerebral infarctions); (2) patients who had experienced the onset of cognitive decline within the past 1–6 months; (3) patients who were between 50 and 85 years of age; (4) patients with education levels of 6 years or more; (5) patients with MoCA scores less than 26; and (6) patients with informed consent forms signed by either themselves or their family members.

The exclusion criteria were as follows: (1) patients with preexisting dementia or cognitive impairments secondary to Alzheimer's disease or other diseases; (2) patients with language, auditory, and/or visual impairments who could not complete the MoCA; and (3) patients who were unable to complete baseline testing or had contraindications to TMS.

The trial suspension criteria were as follows: (1) participants with deteriorating conditions or serious complications that rendered them unable to continue to participate; (2) participants who did not follow the study's regulations; (3) patients who could not tolerate TMS therapy; and (4) participants who requested to leave the study.

### 2.2 TMS Protocol

A figure‐eight‐shaped coil was positioned over the DLPFC, with the coil tangent to the intersection point of the line connecting the two tragus and the midline of the head. During the initial rTMS session, the motor threshold (MT) was determined by recording motor‐evoked potentials (MEPs) in the muscle belly of the abductor pollicis brevis. In accordance with the 2012 International Federation of Clinical Neurophysiology guidelines, stimulation intensity began at 35% of the maximum output intensity (MOI) and was increased in 5% increments until each TMS pulse reliably elicited an MEP with an amplitude of >50 µV. Stimulation intensity was then decreased in 1% increments to identify the lowest intensity capable of inducing an MEP amplitude of >50 µV in at least five out of 10 trials. This value was defined as the resting motor threshold (RMT).

In this study, the stimulation intensity was set to the maximum level tolerable to the patient, not exceeding 120% of the RMT, and with an intensity ranging from 60% to 120% of the RMT. Stimulation was set to a frequency of 5 Hz, and we delivered a total of 1200 pulses over sessions conducted 5 days a week for four consecutive weeks.

### 2.3 Routine Rehabilitation

Routine poststroke rehabilitation includes personalized cognitive function training and limb function training. Cognitive function training focuses on enhancing attention, executive function, and memory function. Training sessions were performed twice a day, with each session lasting approximately 30 min, and were conducted 5 days a week for a total of 4 weeks.

### 2.4 Cognitive Function Assessments

All participants underwent baseline Montreal Cognitive Assessment (MoCA) (Nasreddine et al. 2005; Xu et al. 2022) within 3 days after admission.

The MoCA includes visuospatial, executive, naming, memory, attention, language, abstraction, delayed recall, and orientation components. A score of less than 26 indicates cognitive impairment.

### 2.5 Statistical Analysis

We used GraphPad Prism 9.4.1 (GraphPad Software, Inc., San Diego, CA, USA) for statistical analysis and graphing. Normally distributed data were expressed as means ± standard deviations ($\bar{x}$ ± s). Nonnormally distributed data were expressed as the median (M) and interquartile range (IQR). We compared baseline characteristics, including sex, diagnosis (cerebral infarction [CI] and intracerebral hemorrhage [ICH]), time of onset, site (anterior circulation [AC] and posterior circulation [PC]), and educational level, between the TMS and control groups. Fisher's exact test (for percentages) was performed to evaluate any between‐group differences in sex, diagnosis, and stroke site. Between‐group comparisons were assessed with unpaired *t*‐tests or Mann–Whitney tests, while within‐group comparisons were analyzed with paired *t*‐tests or Wilcoxon tests. A *p*‐value of <0.05 was considered statistically significant.

## 3 Results

Baseline characteristics were compared between the two groups. There were no between‐group differences in age, sex, diagnosis, time of onset, site, or educational level (Table 1). There were also no between‐group differences on the lesion side (Table 2).

**TABLE 1:** Baseline characteristics in the TMS and control groups.

Group	Age	Site		Sex (*n*)		Diagnosis		Time of onset	Educational level
	(years)	AC/PC		Male	Female	CI	ICH	(days)	(years)
TMS	69.62 ± 9.32	10	3	8	5	11	2	99.00 (46.00, 125.00)	12.00 (7.50, 15.00)
Control	70.40 ± 11.20	15	5	13	7	15	5	44.00 (30.50, 87.75)	7.50 (6.00, 9.00)
*t*/*U*	0.2095	—		—		—		80.5	85.50
*p*	0.8354	>0.9999		>0.9999		0.6756		0.0690	0.0896

*Note*: A *p*‐value of <0.05 indicates statistical significance.

Abbreviations: A, anterior circulation; CI, cerebral infarction; ICH, intracerebral hemorrhage; P, posterior circulation; TMS, transcranial magnetic stimulation.

**TABLE 2:** Baseline characteristics in the left‐sided and right‐sided groups.

Group	Age	Site		Sex (*n*)		Diagnosis		Time of onset	Educational level
	(years)	AC/PC		Male	Female	CI	ICH	(days)	(years)
Left‐sided	71.29 ± 12.01	6	1	4	3	6	1	77.00 ± 42.65	13.00 ± 5.10
Right‐sided	67.67 ± 5.20	4	2	4	2	5	1	95.00 ± 45.67	10.00 ± 3.63
*U*	17.50	—		—		—		16.00	13.50
*p*	0.6556	0.5594		>0.9999		>0.9999		0.5338	0.3048

*Note*: A *p*‐value of <0.05 indicates statistical significance.

Abbreviations: A, anterior circulation; CI, cerebral infarction; ICH, intracerebral hemorrhage; P, posterior circulation.

**FIGURE 1:**
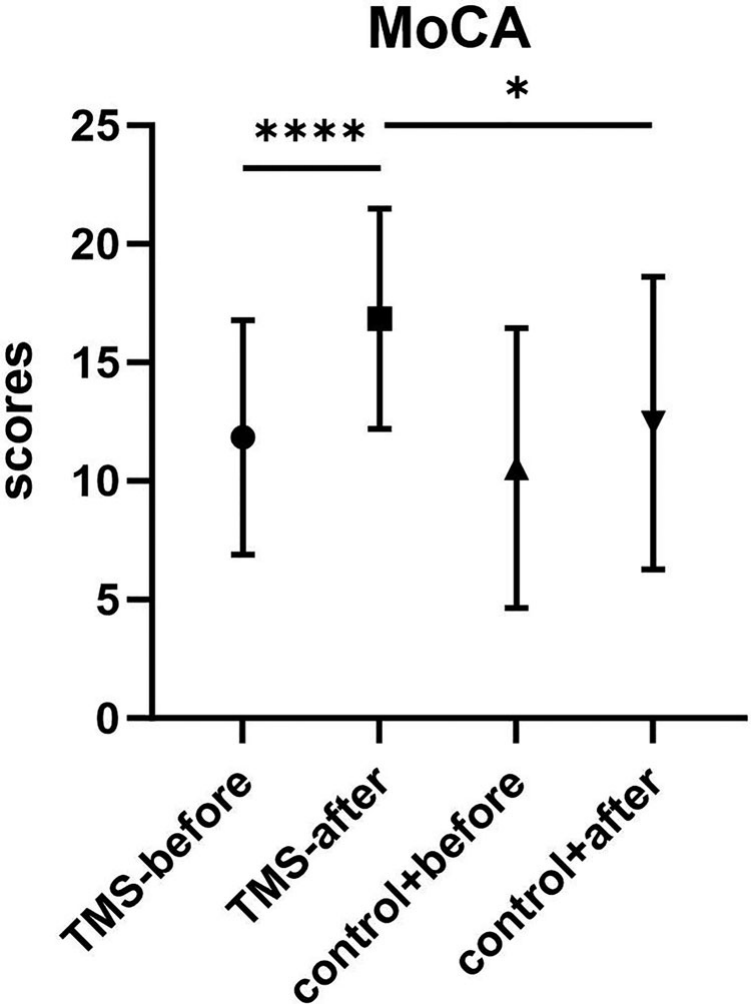
MoCA scores in the TMS group before and after treatment and in the control group. Scores in the TMS group were significantly higher after treatment than before treatment and compared with the control group (*p* < 0.05). MoCA, Montreal Cognitive Assessment; TMS, transcranial magnetic stimulation.

MoCA scores in the TMS group increased significantly after treatment compared with before treatment (*p* < 0.0001). MoCA scores in the TMS group also increased significantly after treatment compared with the control group (*p* = 0.0359) (Figure 1).

**FIGURE 2:**
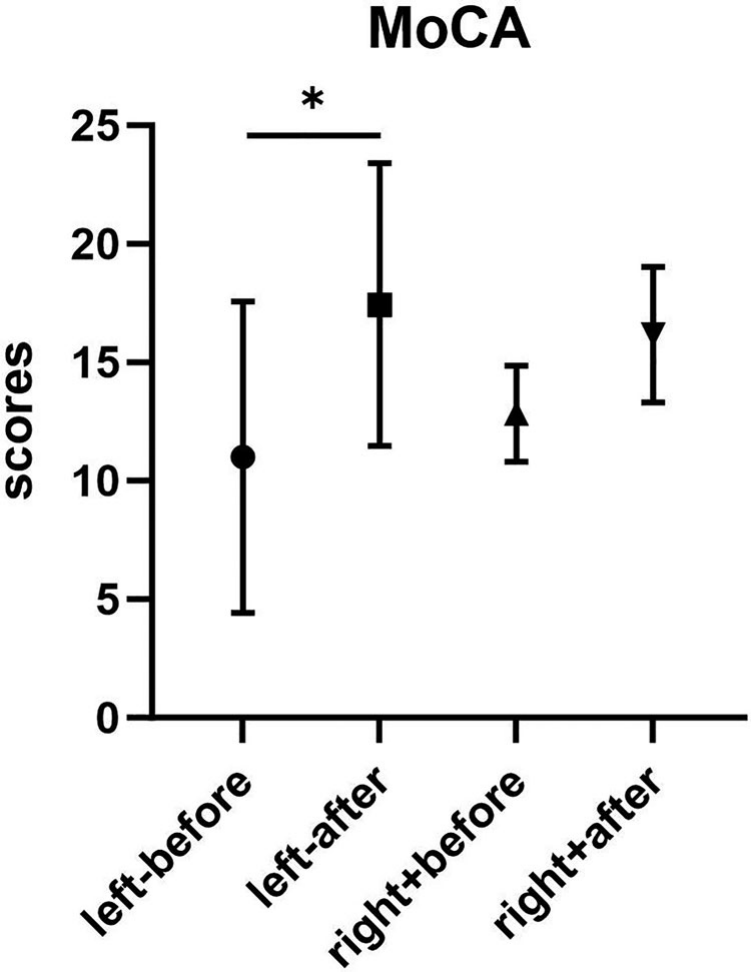
MoCA scores in the TMS group stratified by lesion side (left and right) before and after treatment. A significant increase was observed only in patients with left‐sided lesions after treatment (*p* < 0.05). MoCA, Montreal Cognitive Assessment.

In the TMS group (including those with left‐sided lesions and those with right‐sided lesions), MoCA scores increased significantly only in those with left‐sided lesions after treatment compared with before treatment (*p* = 0.0156) (Figure 2).

## 4 Discussion

Our results showed that MoCA scores significantly increased after treatment in the TMS group relative to the control group and increased significantly in both the TMS and control groups after treatment compared with before treatment. In the TMS group (including those with left‐side and right‐side lesions), MoCA scores significantly increased only in patients with left‐side lesions after treatment compared with before treatment. However, there were no significant between‐group differences in scores after treatment—the results were similar whether or not the lesion was ipsilateral to the stimulation site.

Strokes can impair both global cognitive functioning and cognitive subdomains such as executive function. TMS over the DLPFC has been shown to effectively address cognitive impairments after stroke. A recent meta‐analysis showed that this treatment significantly enhances attention, language, memory, and visuospatial functions (Zhao et al. 2024). Another systematic review and meta‐analysis demonstrated the positive effects of combined TMS and cognitive function training on global cognitive function, executive function, and working memory (Gao et al. 2023). However, major TMS parameters such as stimulation frequency, stimulation site, and stimulation duration have varied significantly across past studies.

High‐frequency TMS applied to the left DLPFC is the most common TMS treatment for cognitive impairment. The left and right cortical hemispheres have significant structural and functional differences (Mancuso et al. 2019). Gonzalez Alam et al. (2022) showed that the left cerebral hemisphere is predominantly associated with semantic cognition and language processing, while the right cerebral hemisphere is responsible for visual reasoning and attention‐related functions. In patients with left cerebral hemisphere strokes and aphasia, nonverbal cognitive impairments, such as executive dysfunction and naming deficits, appear to be more pronounced (Meier et al. 2022). Among all of the cortical regions, the DLPFC plays a key role in regulating numerous aspects of cognitive processing, including attention (Stonsaovapak et al. 2020), working memory (Vékony et al. 2018), decision‐making (Obeso et al. 2021), and inhibitory control (McNeill et al. 2018; Chen et al. 2021). Previous studies have shown that the DLPFC is primarily involved in top‐down attentional modulation and working memory (Lowe et al. 2018), in addition to regulating automatic behavioral patterns (Xia et al. 2021). High‐frequency TMS can alter synaptic plasticity, increase cortical excitability, and consequently affect neural functions in both local and distant brain regions (Lefaucheur et al. 2020). Thus, the left DLPFC is commonly selected as a target for TMS treatment. A recent review and meta‐analysis suggested that excitatory stimulation over the left DLPFC can significantly improve global cognitive functioning (Han et al. 2023). In the present study, we assessed global cognitive functioning using MoCA scores and found that it was significantly improved after 5‐Hz TMS therapy was applied over the left DLPFC. This finding was consistent with previous studies and further supports the positive impacts of 5‐Hz TMS on cognitive functioning. However, we did not evaluate the effects of TMS on cognitive subdomains, such as attention, executive function, or memory.

There are several potential mechanisms that could underlie functional recovery after stroke. Previously proposed models include the vicariation model, the interhemispheric inhibition model, and the bimodal balance‐recovery model, which is based on the concept of “structural reserve” (Di Pino et al. 2014). Soleimani et al. (2023) showed that early improvements in cognitive function after strokes rely on compensatory mechanisms mediated by the contralateral hemisphere. Over time, brain function in the affected hemisphere progressively recovers and helps further restore cognitive abilities (Soleimani et al. 2023). In the bimodal balance‐recovery model, a higher structural reserve is conducive to the interhemispheric competition model. Specifically, inhibiting the contralateral hemisphere can reduce its suppressive influence on the affected hemisphere, which facilitates functional recovery. Conversely, when the structural reserve is low, the variation model is predominant. In this case, enhancing contralateral hemisphere activity may help it restore cognitive or motor functions. Previous studies have found that low‐frequency (1 Hz) TMS applied to the contralateral cerebral hemisphere can partially restore the dynamic functional network (Qin et al. 2021).

In our study, we attempted to explore whether there are any differences in cognitive function improvement after a 5‐Hz protocol to the left DLPFC for different lesion sides in stroke. However, we did not find any significant between‐group differences in functional recovery between patients who had right‐ versus left‐sided lesions after the therapy. This may be because TMS stimulation to the left DLPFC is effective regardless of whether the lesion is ipsilateral to the stimulation site. On the other hand, our study only involved neuropsychological assessments while contemporary research in this field is increasingly employing multimodal assessments, combining behavioral scales with neuroimaging techniques like fNIRS or EEG to explore underlying neural mechanisms. In addition, all of these cortical recovery models were initially developed for motor functional recovery, not cognitive functional recovery. The brain regions and circuits associated with cognitive function are far more extensive and intricate than those associated with motor function. Thus, whether these models can be extrapolated to study cognitive functional recovery remains unclear. The precise stimulation site and the optimal parameters—including frequency, intensity, duration, and coil orientation—remain to be systematically investigated.

Despite these promising results, our study has several limitations. First, it was a retrospective study with a small sample size (*n* = 33), which affects the generalizability of our results. Second, we did not have follow‐up data and, therefore, could not evaluate possible changes in cognitive functioning over the course of the recovery period. Third, in the TMS group, we explored the impact of the lesion side on treatment efficacy under the condition that all patients received left‐sided DLPFC TMS therapy. There were several inherent limitations to this approach, and ideally, studies on the effects of TMS on either the healthy or affected hemisphere should not be restricted to a single target site. Fourth, our participants had poststroke recovery durations of between 1 and 6 months when they were enrolled in our study, which is a relatively broad time span. This variability may have contributed to the considerable heterogeneity we observed in cortical excitability changes, which could have influenced treatment outcomes. Fifth, our only evaluative outcome was the MoCA score, which reflects overall cognitive functioning. Future studies could incorporate additional assessment tools targeting specific cognitive domains, such as executive functioning and attention, which would enable a more comprehensive evaluation of treatment efficacy.

In summary, we found that high‐frequency (5 Hz) TMS applied to the left DLPFC significantly enhanced overall cognitive functional recovery in poststroke patients. We also found that when we applied 5‐Hz TMS to the left DLPFC, its effects were similar regardless of whether or not the lesion was ipsilateral to the stimulation site. These results confirm the role of the left DLPFC as a classical target for improving cognitive function in the subacute poststroke recovery phase.

## Author Contributions

**Hui‐xian Yu**: funding acquisition, formal analysis, writing – original draft. **Qian Diao**: data curation, funding acquisition. **Yu‐juan Han**: data curation. **Bo Wei**: data curation. **Pei Dai**: methodology, writing – review and editing, formal analysis.

## Funding

This research was supported by the National Natural Science Foundation of China (grant number: 82302844) and the Beijing Municipal Administration of Hospitals Incubating Program (grant number: PX2023074).

## Ethics Statement

This study was conducted in accordance with the Declaration of Helsinki (1964) and was approved by the Ethics Committee of Beijing Xiaotangshan Hospital. All participants provided signed and informed consent. The ethics review number is 2023‐17.

## Conflicts of Interest

The authors declare no conflicts of interest.

## Data Availability

The raw data supporting the conclusions of this article will be made available by the authors on request. The Chinese version of the MoCA assessment scale used in this manuscript is currently available for open use.
